# Delayed Onset of Symptoms After a Rattlesnake Bite in a Renal Transplant Patient: A Case Report

**DOI:** 10.5811/cpcem.1280

**Published:** 2023-12-06

**Authors:** Thomas A. Rogers, John Herrick

**Affiliations:** Christus Spohn Health System, Department of Emergency Medicine, Corpus Christi, Texas

**Keywords:** *case report*, *envenomation*, *transplant*, *immunosuppression*, *snakebite*

## Abstract

**Introduction:**

The United States is home to two major families of venomous snakes, Crotalids and Elapids. The Crotalid family, also known as pit vipers, is well known for being among the most frequent causes of snakebites reported. Crotalid envenomation can present with local findings, hematologic toxicity, and systemic toxicity. Identification of envenomated patients is key to determining who needs antivenom. Most sources recommend an observation period of six to eight hours after the snakebite to determine whether the bite was “dry” or the patient was exposed to venom.

**Case Report:**

We present the case of a 33-year-old patient with a history of renal transplantation who had delayed onset of symptoms of envenomation 18 hours after an initial emergency department observation. The patient did well after a course of antivenom and was discharged on hospital day three.

**Conclusion:**

The patient’s immunosuppressive regimen may have delayed the onset of clinical symptoms, thus delaying treatment. To the best of our knowledge, this is the first case reported of a patient presenting with a delayed onset of initial snakebite envenomation symptoms.

Health Population Research Capsule What do we already know about this issue?

*To the best of our knowledge, this is the first instance of the occurrence of a delayed reaction to a snakebite in a renal transplant patient or any patient undergoing immunosuppression.*

What was the research question?

*The question in our case was, “Is there a physiological reason for patient’s undergoing immunosuppression to have a delayed response to crotalid venom?”*

What was the major finding of the study?

*This case report found that the patient underwent an appropriate duration of observation at a rural emergency department but developed symptomatic envenomation from the bite approximately 18 hours later.*

How does this improve population health?

*This case is a reminder that immunosuppressed patients can have delayed onset of physical exam findings and may present differently in cases of acute evenomation. Furthermore, it is a reminder to involve specialists in the care of the majority of transplant patients presenting to the emergency department.*

## INTRODUCTION

Venomous North American snakes come from two families: Elapidae and Viperidae, the latter of which can be divided into two subfamilies: Crotalinae and Viperinae. Snakes from the Crotalinae subfamily are those that are found in North America, whereas snakes from Viperinae are found in Europe, Asia, and Africa.^1^ The Crotalid family of snakes includes three genera: Crotalus and Sistrurus (rattlesnakes), as well as Agkistrodon (water moccasins and copperheads).^1^ Reported cases of venomous snakebites in the United States range from 5,000–10,000 cases per year.^1^ ^,^ ^2^ Crotalinae envenomation syndromes present with three major categories of symptoms: local swelling, hematologic toxicity, and systemic toxicity.^3^ Local reactions include swelling at the bite site, which can progress to hemorrhagic bleb formation and spreading of the swelling proximal to the bite. Hematologic toxicity is generally due to hypofibrinogenemia and thrombocytopenia.^4^ Systemic toxicity is the shock state that may occur at any point in the hours after envenomation.

Treatment of these bites starts with the basics of trauma care and resuscitation. Assuming the patient is asymptomatic, the common teaching is to observe the patient for six to eight hours after the bite with a full laboratory workup and physical exam repeated during the observation time frame.^5^ Of all patients who are bitten, however, approximately 80% will require antivenom, which in the US is either ANAVIP or CroFab.^5^ Cases of delayed hematologic complications are described in the literature after antivenom administration, but delayed presentation of envenomation is less well described.^6^ We present the case of a patient with a history of a renal transplant who presented with delayed onset of symptoms of envenomation after initial emergency department (ED) observation.

## CASE REPORT

A 33-year-old male patient with a past medical history of hypertension, type II diabetes mellitus, and renal transplant presented to an outside ED at approximately 7 pm, one hour after suffering a rattlesnake bite to his right third digit. The patient reported that he was prescribed tacrolimus 10 milligrams (mg) daily and prednisone 10 mg daily for rejection prophylaxis. His initial vital signs were within normal limits. Laboratory workup showed a prothrombin time of 10.4 seconds (s) (reference range 11–13.5 s), international normalized ratio 1.0 (reference range 0.8–1.1), a fibrinogen of 240 mg per deciliter (dL) (200–400 mg/dL), and platelets of 192,000 per microliter (μL) (150,000–450,000/μL). The patient received three doses of hydromorphone intravenous (IV) injection for pain control and underwent serial measurements of his digit across the span of his seven-hour observation recommended by the Unified Treatment Guidelines for snakebites.^5^

Laboratory workup was repeated at 9:15 pm, and values were unchanged from his initial measurements aside from a fibrinogen of 183 mg/dL; however lab tests were not repeated immediately prior to discharge. The initial emergency physician administered 1 mg ceftriaxone and discharged the patient with a prescription for oral analgesia and an antibiotic at 1 am. The documentation at the time of discharge noted that the patient’s pain was under control and that his finger had no evidence of injury aside from the puncture wound.

**Figure 1.**
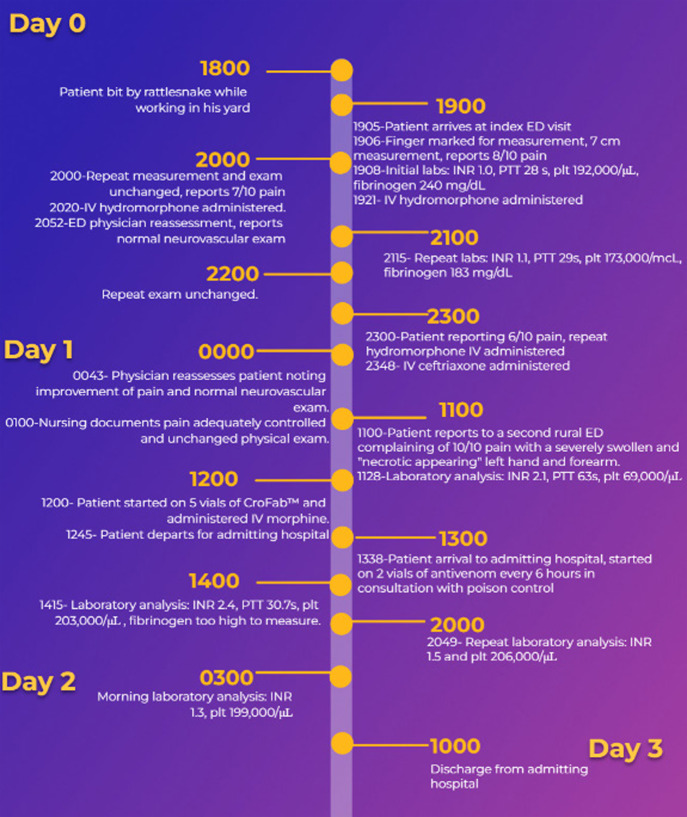
Timeline of the care and evaluation of an immunocompromised patient suffering a snakebite. *IV*, intravenous; *INR*, international normalized ratio; *PTT*, partial thromboplastin time; *plt*, platelets.

At 11 am later that day, the patient presented to a second ED with complaints of significant swelling and discoloration of his right arm. The physician documentation at that time was that the third digit of his right hand appeared necrotic. His workup at that time was significant for platelets of 69,000 per μL and an INR of 2.1. The patient was started on five vials of CroFab antivenom and was transferred to our facility at 1 pm. On arrival to our facility, the patient was noted to have a PT of 22.9s and INR of 2.4 and his platelets had rapidly improved to 203,000/μL. In consultation with the South Texas Poison Control Network, the emergency physician and admitting physician started the patient on maintenance dosing of antivenom with two vials every six hours and initiated laboratory monitoring until resolution of his coagulopathy. The admitting physician also started the patient on IV clindamycin 900 mg every eight hours. A timeline of the events of the patient’s care is below (Figure 1).

He was then admitted to the general wards for two days. His coagulopathy and symptoms completely resolved by hospital day three. The patient was discharged home with primary care follow-up and a prescription for oral clindamycin and analgesia.

## DISCUSSION

The most well-known treatment algorithms recommend an observation period of six to eight hours after the initial snakebite before discharge.^5^ Our patient underwent observation of approximately seven hours after the initial bite, and his only indication that he may have been envenomated was pain, which resolved prior to discharge. Serial measurements from his first ED visit were consistent and unchanging throughout his stay. The classic envenomation syndrome with swelling and coagulopathy occurred 18 hours after the bite, well outside the recommended window of observation. We believe this may have been an effect modulated by his immunosuppressive regimen of tacrolimus and prednisone. Snake venoms contain several enzymes meant to assist the animal in the capture and consumption of prey. One component, snake venom metalloproteinases, assists in tissue necrosis at the site of the bite by activating tumor necrosis factor, which in turn results in cytokine release, leukocyte migration, neutrophil recruitment and degranulation, and macrophage differentiation.^1^ ^,^ ^3^ ^,^ ^4^ ^,^ ^7^ ^,^ ^8^ Tacrolimus and prednisone are both known to modulate inflammatory responses due to cytokine release and decrease the chances of acute rejection.^9^ ^–^ ^11^

We theorize that these immunosuppressive effects may have blunted the physical exam findings of acute envenomation, resulting in a delayed presentation of our patient with envenomation syndrome. There are some limitations to this report. The most important of which is that this patient initially reported to two different, remote EDs, which are generally staffed by non-emergency medicine trained physicians. That being said, the documentation at that time suggests that the patient was asymptomatic and pain free at the time of discharge. We also contend that patients with immunosuppression syndromes should likely undergo prolonged observation due to the chance of delayed reactions to Crotalid envenomation.

## CONCLUSION

We present the case of a young man who had delayed onset of rattlesnake envenomation syndrome after the traditional observation period in the ED. The patient had a complicating medical history of renal transplantation, which may have caused his delayed presentation. The patient was discharged on hospital day three in good condition. To the best of our knowledge, this is one of the only cases of delayed presentation of a snakebite envenomation syndrome presented in the literature.
